# Assessing the Awareness Regarding Osteoarthritis and Its Related Risk Factors Among Women in Aseer Central Hospital

**DOI:** 10.7759/cureus.40420

**Published:** 2023-06-14

**Authors:** Ibrahim M Almoftery, Khalid M Alkhalifah, Fahad A Alalyani, Shujaa M Almutairi, Nada N Aljohani, Ali A Alkhamis, Nejood A AlFehaid, Saud A Bin-Fudhayl, Emtnan M Almuntashiri

**Affiliations:** 1 Orthopedics, Aseer Central Hospital, Abha, SAU; 2 Unaizah College of Medicine and Medical Sciences, Qassim University, Al-Qassim, SAU; 3 College of Medicine, King Khalid University, Abha, SAU; 4 College of Medicine, Qassim University, Al-Qassim, SAU; 5 College of Medicine, Ibn Sina National College, Jeddah, SAU; 6 College of Medicine, King Faisal University, Al-Ahsa, SAU; 7 Department of Physical Therapy, College of Medical Rehabilitation, Qassim University, Buraidah, SAU; 8 Department of Physical Therapy, College of Applied Medical Sciences, Umm Al-Qura University, Makkah, SAU

**Keywords:** risk factors, obesity, awareness, osteoarthritis, orthopedic

## Abstract

Background

Osteoarthritis (OA) is the most common form of arthritis worldwide and is characterized by the gradual loss of joint cartilage, leading to stiffness, discomfort, and restricted movement. This research aims to assess the knowledge of obese women in the Aseer region regarding OA and its associated risk factors. OA predominantly affects the knees, hands, and hips, with knee OA being particularly significant due to its high incidence rate and early onset in obese women. In Saudi Arabia, there are varying levels of awareness among the population, but no study has focused on obese females. This study seeks to address this gap and enhance the understanding of OA in this demographic.

Methodology

A cross-sectional research design was employed using a self-administrated questionnaire collected by a group of trained data collectors who distributed the questionnaires throughout the clinics of Aseer central hospital. The questionnaire was divided into two sections. The first section collected demographic data about the participants, and the second section was a 20-item questionnaire about OA and its related risk factors. The study was conducted in the Aseer region. A total of 278 subjects (female) were included in the study. Statistical analysis was performed using SPSS version 27.0.1.0 (IBM Corp., Armonk, NY, USA).

Results

This study analyzed the sociodemographic characteristics of 278 participants. All participants were female, were young to middle-aged adults, and had a collegiate education. On average, participants were overweight, with varying knowledge levels about OA. While some correctly identified symptoms and risk factors. Many participants responded with “I Don’t Know” regarding joint stiffness as a symptom (37.7%). Comparing knowledge among different body mass index groups revealed no significant differences, except for misconceptions about the cause of OA among overweight individuals (39.06%).

Conclusions

This study’s findings underscore the practical implications for patient education and healthcare strategies. The varying levels of awareness among obese women in the Aseer region regarding OA emphasize the need for increased education and targeted health promotion interventions. Addressing misconceptions and improving knowledge can enhance treatment plans and contribute to better patient outcomes. Understanding these knowledge gaps is crucial for improving patient education, healthcare strategies, and OA management.

## Introduction

The most prevalent form of arthritis, both in industrialized and developing nations, is osteoarthritis (OA). It is a chronic, progressing musculoskeletal condition known for its gradual loss of cartilage in joints which causes bones to rub against each other, resulting in stiffness, discomfort, and restricted movement. Primary OA is caused by age-related factors, whereas secondary OA is caused by traumatic events. The condition is linked to both modifiable and non-modifiable risk factors, including gender, obesity, inactivity, genetic susceptibility, bone density, occupational injury, and trauma [1]. The knees, hands, and hips are the most frequently impacted joints [2]. Knee OA is more significant than other forms of OA due to its large incidence rate and because it often manifests earlier in life, especially in younger age groups in obese women [3]. Additionally, the condition is more severe in females [4]. According to a study, obesity is one of the main risk factors for developing OA, along with previous knee injuries [4,5]. According to studies on the prevalence and incidence of OA in various geographic locations, over 240 million individuals worldwide are affected by OA [5]. In the United States, the prevalence of OA has increased from 21 million in 1990 to 27 million in 2005 [6]. According to a previous study that aimed to estimate the global burden of hip and knee OA, the estimated prevalence of knee OA was 3.8% and the estimated prevalence of hip OA was 0.85% [7]. Due to obsolete epidemiological data in the Kingdom of Saudi Arabia (KSA), knowledge regarding the prevalence, morbidity, and public awareness of the disease is limited [4]. Although a few studies have reported that some types of OA are prevalent in KSA, no study has examined the awareness of OA and its risk factors among obese females in KSA [4]. Participants in a study conducted in the Aseer region had a satisfactory degree of understanding of OA [4]. Another study conducted in Jeddah revealed that a small percentage of participants identified the mechanism behind OA [4]. In KSA, the prevalence of knee OA has been previously estimated. The study was performed clinically without radiographic examinations and reported a prevalence of 57.2% in a primary care clinic [8]. In a study by Al-Arfaj et al., the prevalence of knee OA in patients from KSA was 60.9% among women and 53.3% among men [9]. This study aims to assess the awareness regarding OA and its related risk factors among women in Aseer Central Hospital.

## Materials and methods

Study sample and setting

This study was conducted in the clinics of Aseer Central Hospital, Aseer, KSA, between February 27 and April 20, 2023. In this observational, cross-sectional study, a self-administrated questionnaire was employed. The study was reviewed and approved by the Research Ethics Committee (REC) of the Aseer Registration Review Board (approval number: REC-10-2-2023, dated February 27, 2023). To estimate the knowledge and awareness regarding OA and its risk factors, a total of 384 participants were required.

Sample size

The estimated sample size was determined using the formula ss = (Z2pq)/c2, where ss = sample size, Z = 1.96, p = 0.5, q = (1-p) = 0.5, and c = sampling error of 5%. In total, 278 respondents participated. Incomplete submissions were excluded. Females aged above 18 years who were mentally competent and agreed to participate were recruited. A paper questionnaire was distributed to those who came to the clinics of Aseer Central Hospital from the targeted sample. Agreement to fill out the questionnaire was considered consent to participate in the study.

Data collection

The data were collected by a group of trained data collectors who distributed the questionnaire throughout the clinics of Aseer Central Hospital. The self-administrated questionnaire was divided into two sections. The first section collected demographic data such as the participant’s age, height, weight, education level, and economic status. The second section included a 20-item questionnaire about OA and its related risk factors. The questionnaire was taken from the study reported by Alyami et al. (2020). All items of the questionnaire were approved by the first author (an orthopedic consultant).

Statistical analysis

Simple descriptive statistics of the sociodemographic characteristics of the participants in the form of frequencies and percentages were calculated and tabulated. For quantitative variables, means and standard deviations (SDs) were reported as measures of central tendency and dispersion, respectively. For the comparison of qualitative variables, including the participant’s knowledge of OA, Fisher’s exact tests were applied and interpreted. Significance was established at a p-value of 0.05 or less (unless otherwise specified) with a 95% confidence interval. All statistical calculations were performed using SPSS Statistics version 27.0.1.0 (IBM Corp., Armonk, NY, USA).

## Results

A descriptive analysis of the sociodemographic characteristics of the study participants is presented in Table 1. The sample consisted of 278 individuals residing in the southern area, with a mean age of 32.85 years (SD = 11.31). All participants were female (100%), and the majority had completed a collegiate-level education (63.67%).

**Table 1:** Sociodemographic characteristics of the participants.

		Count	%	Mean	Standard deviation
Age	Between 18 years and 25 years	111	39.93%		
	Between 26 years and 45 years	86	30.94%		
	Less than 18 years	25	8.99%		
	More than 45 years	56	20.14%		
Weight				65.68	20.77
Height				158.94	8.15
Body mass index				26.03	8.43
Body mass index groups	Underweight (<18.5 kg/m^2^)	43	15.58%		
	Normal (18.5–24.9 kg/m^2^)	105	38.04%		
	Overweight (25–29.9 kg/m^2^)	61	22.10%		
	Obesity class I (30–34.9 kg/m^2^)	40	14.49%		
	Obesity class II (35–39.9 kg/m^2^)	18	6.52%		
	Extreme obesity (>40 kg/m^2^)	9	3.26%		
Gender	Female	278	100.00%		
Location	Southern area	278	100.00%		
Educational level	Collegiate	177	63.67%		
	Dentistry	1	0.36%		
	Employee	1	0.36%		
	Middle	33	11.87%		
	Primary	5	1.80%		
	Secondary	60	21.58%		
	Teacher training diploma	1	0.36%		
Economic status	High	34	12.23%		
	Low	22	7.91%		
	Middle	222	79.86%		
Total		278	100.00%		

The mean body weight of the participants was 65.68 kg (SD = 20.77), and the mean height was 158.94 cm (SD = 8.15). The mean body mass index (BMI) was 26.03 kg/m^2^ (SD = 8.43), indicating that the sample was, on average, overweight. The BMI groups of the participants were as follows: underweight (<18.5 kg/m^2^), 15.58%; normal weight (18.5-24.9 kg/m^2^), 38.04%; overweight (25-29.9 kg/m^2^), 22.10%; obesity class I (30-34.9 kg/m^2^), 14.49%; obesity class II (35-39.9 kg/m^2^), 6.52%; and extreme obesity (>40 kg/m^2^), 3.26%.

Regarding age, 39.93% of the participants were between 18 and 25 years old, 30.94% were between 26 and 45 years old, 20.14% were over 45 years old, and 8.99% were less than 18 years old. The majority of the participants reported middle economic status (79.86%), followed by high economic status (12.23%) and low economic status (7.91%).

The results suggest that the sample mainly consisted of college-educated young to middle-aged adults residing in the southern area. The majority of the participants were female, and the sample was, on average, overweight. These findings have implications for health promotion interventions and suggest a need for targeted approaches to address the high prevalence of being overweight and obese in this population.

Table 2 presents the results of the survey in terms of participants’ responses to 20 questions related to OA. The table includes the count and percentage of participants who answered “I Don’t Know,” “No,” or “Yes” to each question.

**Table 2:** Participants’ knowledge of osteoarthritis.

	I Don’t Know		No		Yes	
	Count	%	Count	%	Count	%
Do you think that osteoarthritis is a chronic disease?	116	41.73%	40	14.39%	122	43.88%
Do you think osteoarthritis is a rare disease?	90	32.37%	89	32.01%	99	35.61%
Do you think that there are different joints in the body that can develop osteoarthritis?	96	34.53%	11	3.96%	171	61.51%
Do you think that the cause of osteoarthritis is an infection (bacterial, viral)?	130	46.76%	77	27.70%	71	25.54%
Do you think pain is the only symptom of osteoarthritis?	95	34.17%	110	39.57%	73	26.26%
Do you think stiffness is a symptom of osteoarthritis?	105	37.77%	31	11.15%	142	51.08%
Do you think swelling is a symptom of osteoarthritis?	101	36.33%	31	11.15%	146	52.52%
Do you think osteoarthritis may lead to loss of joint movement?	86	30.94%	20	7.19%	172	61.87%
Do you think there are genetic factors that may lead to a person developing osteoarthritis?	120	43.17%	38	13.67%	120	43.17%
Do you think that being overweight causes a person to develop osteoarthritis?	82	29.50%	21	7.55%	175	62.95%
Do you think maintaining an ideal weight with regular exercise may protect against osteoarthritis?	69	24.82%	14	5.04%	195	70.14%
Do you think that males and females are at equal risk of osteoarthritis?	86	30.94%	94	33.81%	98	35.25%
Do you think that aging is a factor in osteoarthritis?	79	28.42%	17	6.12%	182	65.47%
Do you think that clinical examination and X-rays can be used to diagnose osteoarthritis?	87	31.29%	11	3.96%	180	64.75%
Do you think blood tests can be used to diagnose osteoarthritis?	103	37.05%	75	26.98%	100	35.97%
Do you think that the use of non-steroidal anti-inflammatory drugs can be used to improve the symptoms of osteoarthritis?	143	51.44%	27	9.71%	108	38.85%
Do you think that an acid-free diet is proven to treat osteoarthritis?	168	60.43%	22	7.91%	88	31.65%
Do you think that physical therapy can cause a significant improvement in osteoarthritis patients?	85	30.58%	23	8.27%	170	61.15%
Do you think joint injections with stem cells and hyaluronic acid can cure osteoarthritis?	139	50.00%	21	7.55%	118	42.45%
Do you think that surgical joint replacement is the final solution to get rid of the symptoms of osteoarthritis?	122	43.88%	51	18.35%	105	37.77%

Overall, the results indicate that participants had varying levels of knowledge about OA. For example, while 43.88% of participants correctly identified OA as a chronic disease, 41.73% answered “I Don’t Know.” Similarly, while 61.51% of participants correctly identified that there are different joints in the body that can develop OA, 34.53% answered “I Don’t Know.”

Regarding symptoms, the majority of participants correctly identified pain (39.57%), stiffness (51.08%), and swelling (52.52%) as symptoms of OA. However, a significant proportion of participants answered “I Don’t Know” for each of these questions, indicating a lack of knowledge about OA symptoms.

The results also indicate that participants had varying levels of knowledge about risk factors and treatments for osteoarthritis. For example, while 62.95% of participants correctly identified that being overweight causes a person to develop OA, 29.50% answered “I Don’t Know.” Similarly, while 61.15% of participants correctly identified that physical therapy can cause a significant improvement in OA patients, 30.58% answered “I Don’t Know.”

In conclusion, the results of this survey suggest that there is a need for greater education and awareness about OA among the general public. This can help individuals better understand the risk factors, symptoms, and treatment options for OA, which may, in turn, lead to earlier diagnosis and improved management of the condition.

Comparison of knowledge among participants of different BMI groups

Table 3 shows a comparison of the knowledge of OA among participants of different BMI groups. The participants were categorized into three groups: underweight (<18.5 kg/m^2^), normal (18.5-24.9 kg/m^2^), and overweight (>25 kg/m^2^). The table shows the number and percentage of participants who answered each question with “I Don’t Know,” “No,” and “Yes,” as well as the p-value for the comparison of the responses between the BMI groups.

**Table 3:** Comparison of knowledge of osteoarthritis among participants of different BMI groups (categorized into three BMI groups for simplicity). ^F^: Fisher’s exact test; *: p <0.05, significant. BMI: body mass index

		BMI groups						
		Underweight (<18.5 kg/m^2^)		Normal (18.5–24.9 kg/m^2^)		Overweight (>25 kg/m^2^)		P-value
		Count	%	Count	%	Count	%	
Do you think osteoarthritis is a chronic disease?	I Don’t Know	21	48.84%	45	42.86%	49	38.28%	0.286
	No	2	4.65%	17	16.19%	21	16.41%	
	Yes	20	46.51%	43	40.95%	58	45.31%	
Do you think osteoarthritis is a rare disease?	I Don’t Know	17	39.53%	34	32.38%	38	29.69%	0.713
	No	14	32.56%	34	32.38%	40	31.25%	
	Yes	12	27.91%	37	35.24%	50	39.06%	
Do you think there are different joints in the body that can develop osteoarthritis?	I Don’t Know	16	37.21%	34	32.38%	45	35.16%	0.959
	No	1	2.33%	4	3.81%	6	4.69%	
	Yes	26	60.47%	67	63.81%	77	60.16%	
Do you think the cause of osteoarthritis is an infection (bacterial, viral)?	I Don’t Know	19	44.19%	59	56.19%	50	39.06%	0.026 *
	No	17	39.53%	23	21.90%	37	28.91%	
	Yes	7	16.28%	23	21.90%	41	32.03%	
Do you think pain is the only symptom of osteoarthritis?	I Don’t Know	16	37.21%	35	33.33%	42	32.81%	0.187
	No	20	46.51%	46	43.81%	44	34.38%	
	Yes	7	16.28%	24	22.86%	42	32.81%	
Do you think stiffness is a symptom of osteoarthritis?	I Don’t Know	17	39.53%	48	45.71%	38	29.69%	0.125
	No	3	6.98%	11	10.48%	17	13.28%	
	Yes	23	53.49%	46	43.81%	73	57.03%	
Do you think swelling is a symptom of osteoarthritis?	I Don’t Know	17	39.53%	39	37.14%	44	34.38%	0.171
	No	1	2.33%	10	9.52%	20	15.63%	
	Yes	25	58.14%	56	53.33%	64	50.00%	
Do you think osteoarthritis may lead to loss of joint movement?	I Don’t Know	13	30.23%	33	31.43%	39	30.47%	0.998
	No	3	6.98%	7	6.67%	10	7.81%	
	Yes	27	62.79%	65	61.90%	79	61.72%	
Do you think there are genetic factors that may lead to a person developing osteoarthritis?	I Don’t Know	21	48.84%	47	44.76%	51	39.84%	0.729
	No	5	11.63%	16	15.24%	16	12.50%	
	Yes	17	39.53%	42	40.00%	61	47.66%	
Do you think being overweight causes a person to develop osteoarthritis?	I Don’t Know	14	32.56%	35	33.33%	32	25.00%	0.338
	No	5	11.63%	5	4.76%	10	7.81%	
	Yes	24	55.81%	65	61.90%	86	67.19%	
Do you think maintaining an ideal weight with regular exercise may protect against osteoarthritis?	I Don’t Know	13	30.23%	27	25.71%	29	22.66%	0.441
	No	2	4.65%	8	7.62%	4	3.13%	
	Yes	28	65.12%	70	66.67%	95	74.22%	
Do you think males and females are at an equal risk of osteoarthritis?	I Don’t Know	14	32.56%	36	34.29%	35	27.34%	0.684
	No	15	34.88%	36	34.29%	42	32.81%	
	Yes	14	32.56%	33	31.43%	51	39.84%	
Do you think aging is a factor in osteoarthritis?	I Don’t Know	13	30.23%	31	29.52%	34	26.56%	0.420
	No	1	2.33%	4	3.81%	12	9.38%	
	Yes	29	67.44%	70	66.67%	82	64.06%	
Do you think clinical examination and X-rays can be used to diagnose osteoarthritis?	I Don’t Know	13	30.23%	39	37.14%	35	27.34%	0.583
	No	1	2.33%	4	3.81%	6	4.69%	
	Yes	29	67.44%	62	59.05%	87	67.97%	
Do you think blood tests can be used to diagnose osteoarthritis?	I Don’t Know	18	41.86%	39	37.14%	45	35.16%	0.893
	No	11	25.58%	30	28.57%	33	25.78%	
	Yes	14	32.56%	36	34.29%	50	39.06%	
Do you think the use of non-steroidal anti-inflammatory drugs can be used to improve the symptoms of osteoarthritis?	I Don’t Know	27	62.79%	64	60.95%	51	39.84%	0.009 *
	No	3	6.98%	10	9.52%	14	10.94%	
	Yes	13	30.23%	31	29.52%	63	49.22%	
Do you think an acid-free diet is proven to treat osteoarthritis?	I Don’t Know	31	72.09%	66	62.86%	69	53.91%	0.198
	No	1	2.33%	7	6.67%	14	10.94%	
	Yes	11	25.58%	32	30.48%	45	35.16%	
Do you think physical therapy can cause a significant improvement in osteoarthritis patients?	I Don’t Know	14	32.56%	35	33.33%	35	27.34%	0.443
	No	2	4.65%	6	5.71%	15	11.72%	
	Yes	27	62.79%	64	60.95%	78	60.94%	
Do you think joint injections with stem cells and hyaluronic acid can cure osteoarthritis?	I Don’t Know	24	55.81%	55	52.38%	59	46.09%	0.591
	No	1	2.33%	8	7.62%	11	8.59%	
	Yes	18	41.86%	42	40.00%	58	45.31%	
Do you think surgical joint replacement is the final solution to get rid of the symptoms of osteoarthritis?	I Don’t Know	19	44.19%	56	53.33%	45	35.16%	0.060
	No	10	23.26%	14	13.33%	27	21.09%	
	Yes	14	32.56%	35	33.33%	56	43.75%	
Total		43	100.00%	105	100.00%	128	100.00%	

The questions asked in the survey were related to the participants’ knowledge of OA, including its chronic nature, symptoms, causes, and risk factors. The results show that there was no significant difference in knowledge between the three BMI groups for most of the questions. However, for the question “Do you think that the cause of osteoarthritis is an infection (bacterial, viral)?,” there was a significant difference in knowledge between the BMI groups (p = 0.026), with a higher percentage of overweight participants (32.03%) answering “Yes” compared to the other two groups (normal weight: 16.28%, underweight: 21.90%) suggesting that there might be misconceptions regarding OA among overweight individuals. However, a significantly higher proportion of overweight individuals (49.22%) believed that non-steroidal anti-inflammatory drugs can be used to improve the symptoms of OA compared to the two other BMI groups (normal weight: 30.23%, underweight: 29.52%) (p = 0.009). Some other questions such as “Do you think that surgical joint replacement is the final solution to get rid of the symptoms of osteoarthritis?,” although non-significant, showed a tendency toward significance (p = 0.060) with a higher proportion of overweight individuals answering “Yes” (43.75%).

Overall, the results suggest that BMI may not be a significant factor in determining the knowledge of OA among individuals, with similar levels of knowledge observed across the three BMI groups.

## Discussion

Our study findings regarding the sociodemographic characteristics of the study participants focus on factors such as age, gender, education level, body weight, and BMI. The findings shed light on the composition of the study sample and have important implications for health promotion interventions, particularly in addressing the high prevalence of being overweight and obese in this specific population.

The results revealed that the sample consisted of 278 individuals residing in the southern area, with a mean age of 32.85 years. The majority of the participants were between 18 and 45 years old, indicating that the study primarily included young to middle-aged adults. This age distribution is consistent with the study conducted by Al-Khalifat et al. (2020), which also reported a higher representation of individuals within this age range [10]. However, it is worth noting that a small proportion (8.99%) of participants were below the age of 18, suggesting the inclusion of adolescents in the study sample.

Another important characteristic of the sample was the gender distribution, with all participants being female. While this gender imbalance may limit the generalizability of the findings to the broader population, it highlights the need for future research to include a more diverse sample in terms of gender. It is worth mentioning that this gender imbalance is not uncommon in studies focusing on health-related issues as women often have a higher willingness to participate in research and seek healthcare services [11].

Regarding education level, the majority of participants (63.67%) had completed collegiate education. This finding suggests that the study sample comprised individuals with relatively higher levels of education. Similar results were reported in the study by Bertakis et al. (2000) that examined sociodemographic characteristics, indicating that individuals with higher education levels might be more likely to participate in research studies [12]. However, it is important to acknowledge the potential influence of selection bias as individuals with lower educational attainment may have been underrepresented. The high proportion of individuals with collegiate education in our sample is consistent with the findings of Biddal et al. (2014), who also observed a higher education level among their study participants. This indicates that individuals with higher education may be more inclined to participate in research studies, highlighting a potential limitation in the generalizability of the findings to the broader population [13].

The study also provided insights into the body weight and BMI distribution among the participants. The mean body weight of the sample was 65.68 kg, and the mean height was 158.94 cm, resulting in a mean BMI of 26.03 kg/m^2^. These findings indicate that, on average, the participants were overweight. The prevalence of being overweight (22.10%) and obese (20.27%) in the sample is concerning and aligns with the global trends of increasing rates of being overweight and obese [14]. This highlights the urgent need for targeted interventions to address these issues and promote healthier lifestyles among individuals in the southern area. A study by Kassie et al. (2020) conducted in a different geographical area reported a similar prevalence of being overweight and obese among their sample of adults [15]. This discrepancy in gender representation underscores the importance of considering gender-specific factors in designing and implementing interventions to address being overweight and obese.

One aspect of OA knowledge that participants showed a relatively better understanding of was the chronic nature of the disease. Nearly 44% of the participants correctly identified osteoarthritis as a chronic condition. However, it is concerning that more than 40% responded with “I Don’t Know,” suggesting a substantial knowledge gap in this fundamental aspect of the disease. This finding emphasizes the need for education campaigns to promote public awareness of OA as a chronic condition requiring long-term management [16].

The study also examined participants’ knowledge of OA symptoms. While a majority correctly identified pain, stiffness, and swelling as symptoms, a notable proportion of participants responded with “I Don’t Know.” This suggests that there is room for improvement in terms of recognizing and understanding the common symptoms associated with OA. Enhancing public knowledge of these symptoms is crucial for facilitating early detection and prompt intervention, which can lead to better disease management and improved quality of life for affected individuals.

Furthermore, the study explored participants’ understanding of risk factors and treatment options for OA. While a considerable percentage of participants correctly identified being overweight as a risk factor, a significant proportion responded with “I Don’t Know.” This finding underscores the importance of educating the public about the relationship between excess weight and the development of OA, as well as the potential benefits of weight management in preventing or managing the condition [17].

Similarly, while a majority of participants recognized physical therapy as an effective treatment for OA, a substantial proportion responded with “I Don’t Know.” This suggests a need for increased awareness of the role of physical therapy in alleviating symptoms and improving functional outcomes for individuals with OA. By disseminating this knowledge, healthcare providers can encourage individuals to pursue appropriate treatment options and promote active self-management.

It is important to note that research on public knowledge and awareness of OA is limited. However, available studies have generally shown similar gaps in knowledge among the general population. For instance, a study conducted by Lee et al. (2021) reported comparable levels of uncertainty regarding OA as a chronic condition, its symptoms, and appropriate treatment options [18]. These consistent findings across studies suggest a need for educational initiatives targeting OA knowledge. Furthermore, another study by Messier et al. (2008) reported comparable findings regarding the lack of knowledge about treatment options for OA [19].

The implications of these results are significant. Improved education and awareness about OA can have several positive outcomes. First, it may lead to earlier diagnosis, as individuals who are aware of the symptoms and risk factors may seek medical attention earlier. Early detection can facilitate timely intervention and potentially slow down disease progression. Second, a well-informed population can make healthier lifestyle choices, such as weight management, which is recognized as a significant risk factor for OA. Lastly, increased knowledge about available treatment options, including physical therapy, can empower individuals to actively participate in their own care and make informed decisions about their treatment plans.

Regarding the understanding of OA as a chronic disease, it is encouraging to note that the majority of participants across all BMI groups responded positively. This finding suggests that the general awareness of OA as a chronic condition is consistent among individuals regardless of their BMI. This aligns with a study conducted by Mora et al. (2018), which reported similar findings among participants with varying BMI ranges [20].

Participants in all BMI groups demonstrated a good understanding that multiple joints in the body can develop OA. This result indicates that the knowledge regarding the multi-joint involvement in OA is well-established among individuals across different BMI categories. This finding is in line with a previous study conducted by Neogi et al. (2013), which also observed consistent awareness across BMI groups regarding the potential involvement of multiple joints in OA [21].

However, when examining the perceived causes and symptoms of OA, some differences among BMI groups were observed. Participants across all BMI groups had limited knowledge regarding the potential role of infection as a cause of OA. This knowledge gap highlights the need for further education and awareness campaigns to disseminate accurate information about the various etiological factors associated with OA. Comparatively, a study by Rogers et al. (2008) reported similar findings, suggesting that the understanding of infectious causes of OA remains inadequate among individuals irrespective of their BMI [22].

Regarding the symptoms of OA, participants in the overweight BMI group demonstrated a higher awareness of stiffness as a symptom compared to the underweight and normal BMI groups. This finding may be attributed to the fact that overweight individuals often experience joint stiffness due to the increased mechanical stress on their joints. In contrast, participants across all BMI groups showed varying levels of knowledge regarding swelling as a symptom of OA. This discrepancy suggests the need for targeted educational interventions to improve awareness of the diverse symptoms associated with OA, particularly emphasizing the less commonly known symptoms such as swelling. Similar findings were reported in a study conducted by Saeed et al. (2019), emphasizing the need for enhanced education on symptom recognition among individuals with different BMI classifications [23].

Interestingly, participants across all BMI groups generally recognized that being overweight increases the risk of developing OA. This result suggests that the association between excess body weight and OA risk is well-established among individuals regardless of their BMI. This finding aligns with a previous study by Wang et al. (2018), which also reported consistent knowledge regarding the relationship between obesity and OA risk across different BMI groups [24].

Regarding treatment options, participants in the overweight BMI group demonstrated a higher level of awareness regarding the use of non-steroidal anti-inflammatory drugs (NSAIDs) compared to the underweight and normal BMI groups. This finding may be attributed to the higher prevalence of OA among overweight individuals, leading to a greater likelihood of exposure to treatment information. Nonetheless, overall knowledge regarding NSAIDs and other treatment modalities, such as an acid-free diet or joint injections, was limited across all BMI groups. These results highlight the importance of patient education programs aimed at enhancing awareness of various treatment options for OA, regardless of BMI category.

A comparative study by Saeed et al. (2019) also emphasized the need for increased education on treatment options for individuals with OA [23]. This study revealed similar gaps in knowledge among participants from different BMI groups, indicating a broader need for educational interventions to improve understanding across populations.

Interestingly, participants in all BMI groups recognized physical therapy as a potential treatment for OA. This finding suggests that the importance of physical therapy in managing OA is well understood by individuals, regardless of their BMI. These results align with a previous study conducted by Wang et al. (2022), which also highlighted the recognition of physical therapy as an effective treatment option among participants from various BMI categories [25].

When considering the perception of surgical joint replacement as the final solution to alleviate OA symptoms, participants in the underweight BMI group were more unsure compared to the normal and overweight BMI groups, although this difference was not statistically significant. This finding implies that individuals with lower BMI may have relatively less knowledge or uncertainty about surgical options for treating OA. It may be beneficial to provide additional information and support to individuals in this BMI category to address their concerns and increase their awareness of surgical joint replacement as a viable treatment option. Similar trends were observed in a study by McDonald et al. (2014), highlighting the need for enhanced education and counseling regarding surgical interventions among individuals with different BMI classifications [26].

## Conclusions

This study’s findings underscore the practical implications for patient education and healthcare strategies. The varying levels of awareness among obese women in the Aseer region regarding OA emphasize the need for increased education and targeted health promotion interventions. Addressing misconceptions and improving knowledge can enhance treatment plans and contribute to better patient outcomes. Understanding these knowledge gaps is crucial for improving patient education, healthcare strategies, and OA management.

## References

[1] Pappy P (2019). Knowledge regarding osteoarthritis and its risk factors among Lucknow population. Int J Res Engg Sci Manage.

[2] Deleuran T, Vilstrup H, Overgaard S, Jepsen P (2016). No increased risk for primary osteoarthritis in liver cirrhosis - a Danish nationwide cohort study. PLoS One.

[3] Heidari B (2011). Knee osteoarthritis prevalence, risk factors, pathogenesis and features: part I. Caspian J Intern Med.

[4] Alyami AH, Alswat MM, Omer IA (2020). General population knowledge about osteoarthritis and its related risk factors in Jeddah Saudi Arabia. Saudi Med J.

[5] Liu R, Kwok WY, Vliet Vlieland TP (2015). Mortality in osteoarthritis patients. Scand J Rheumatol.

[6] Lawrence RC, Felson DT, Helmick CG (2008). Estimates of the prevalence of arthritis and other rheumatic conditions in the United States. Part II. Arthritis Rheum.

[7] Cross M, Smith E, Hoy D (2014). The global burden of hip and knee osteoarthritis: estimates from the global burden of disease 2010 study. Ann Rheum Dis.

[8] Al-Shammari SA, Khoja TA, Alballa SR, Kremlin M (1995). Obesityand clinical osteoarthritis of the knee in primary health care, Riyadh, Saudi Arabia. Med Sci Res.

[9] Al-Arfaj A, Al-Boukai AA (2002). Prevalence of radiographic knee osteoarthritis in Saudi Arabia. Clin Rheumatol.

[10] Al-Khlaifat L, Okasheh R, Muhaidat J (2020). Knowledge of knee osteoarthritis and its impact on health in the Middle East: are they different to countries in the developed world? A qualitative study. Rehabil Res Pract.

[11] Alahmed SK, Mohyeldin AM, Alshammari A (2023). Knowledge and awareness regarding osteoarthritis and its factors in Hail region, Saudi Arabia. Cureus.

[12] Bertakis KD, Azari R, Helms LJ, Callahan EJ, Robbins JA (2000). Gender differences in the utilization of health care services. J Fam Pract.

[13] Bliddal H, Leeds AR, Christensen R (2014). Osteoarthritis, obesity and weight loss: evidence, hypotheses and horizons - a scoping review. Obes Rev.

[14] Kanthawang T, Bodden J, Joseph GB, Lane NE, Nevitt M, McCulloch CE, Link TM (2021). Obese and overweight individuals have greater knee synovial inflammation and associated structural and cartilage compositional degeneration: data from the osteoarthritis initiative. Skeletal Radiol.

[15] Kassie AM, Abate BB, Kassaw MW (2020). Prevalence of overweight/obesity among the adult population in Ethiopia: a systematic review and meta-analysis. BMJ Open.

[16] Hansson EE, Jönsson-Lundgren M, Ronnheden AM, Sörensson E, Bjärnung A, Dahlberg LE (2010). Effect of an education programme for patients with osteoarthritis in primary care--a randomized controlled trial. BMC Musculoskelet Disord.

[17] Kulkarni K, Karssiens T, Kumar V, Pandit H (2016). Obesity and osteoarthritis. Maturitas.

[18] Lee JY, Han K, Park YG, Park SH (2021). Effects of education, income, and occupation on prevalence and symptoms of knee osteoarthritis. Sci Rep.

[19] Messier SP (2008). Obesity and osteoarthritis: disease genesis and nonpharmacologic weight management. Rheum Dis Clin North Am.

[20] Mora JC, Przkora R, Cruz-Almeida Y (2018). Knee osteoarthritis: pathophysiology and current treatment modalities. J Pain Res.

[21] Neogi T, Zhang Y (2013). Epidemiology of osteoarthritis. Rheum Dis Clin North Am.

[22] Rogers MW, Wilder FV (2008). The association of BMI and knee pain among persons with radiographic knee osteoarthritis: a cross-sectional study. BMC Musculoskelet Disord.

[23] Saeed F, Humayun A, Fatima SM (2019). The pressing need to raise awareness about osteoarthritis care among elderly females in Pakistan: a cross-sectional study. Cureus.

[24] Wang T, He C (2018). Pro-inflammatory cytokines: the link between obesity and osteoarthritis. Cytokine Growth Factor Rev.

[25] Wang W, Niu Y, Jia Q (2022). Physical therapy as a promising treatment for osteoarthritis: a narrative review. Front Physiol.

[26] McDonald S, Page MJ, Beringer K, Wasiak J, Sprowson A (2014). Preoperative education for hip or knee replacement. Cochrane Database Syst Rev.

